# *TREM2* upregulation correlates with 5-hydroxymethycytosine enrichment in Alzheimer’s disease hippocampus

**DOI:** 10.1186/s13148-016-0202-9

**Published:** 2016-04-05

**Authors:** Naiara Celarain, Javier Sánchez-Ruiz de Gordoa, María Victoria Zelaya, Miren Roldán, Rosa Larumbe, Laura Pulido, Carmen Echavarri, Maite Mendioroz

**Affiliations:** Neuroepigenetics Laboratory, Navarrabiomed-Navarra Institute for Health Research (IdiSNA), c/ Irunlarrea, Pamplona, Navarra 31008 Spain; Department of Neurology, Complejo Hospitalario de Navarra, Pamplona, Navarra 31008 Spain; Department of Pathology, Complejo Hospitalario de Navarra, Pamplona, Navarra 31008 Spain; Hospital Psicogeriátrico Josefina Arregui, Alsasua, Navarra 31800 Spain; Present address: Clínica San Miguel, Pamplona, Navarra 31006 Spain

**Keywords:** Gene expression, Alzheimer’s disease, Epigenetics, DNA methylation, *TREM2*, 5-Hydroxymethycytosine

## Abstract

**Background:**

Recent genome-wide association studies revealed *TREM2* rs75932628-T variant to be associated with Alzheimer’s disease (AD) and other neurodegenerative diseases. However, the role that *TREM2* plays in sporadic AD is largely unknown. Our aim was to assess messenger RNA (mRNA) expression levels and DNA methylation profiling of *TREM2* in human hippocampus in AD brain. We measured *TREM2* mRNA levels in the hippocampus in a cohort of neuropathologically confirmed controls and pure AD cases showing no other protein deposits than β-amyloid and phosphorylated tau. We also examined DNA methylation levels in the *TREM2* transcription start site (TSS)-associated region by bisulfite cloning sequencing and further extended the study by measuring 5-hydroxymethycytosine (5hmC) enrichment at different regions of *TREM2* by 5hmC DNA immunoprecipitation combined with real-time qPCR.

**Results:**

A 3.4-fold increase in *TREM2* mRNA levels was observed in the hippocampus of AD cases compared to controls (*p* = 1.1E-05). Interestingly, *TREM2* methylation was higher in AD cases compared to controls (76.2 % ± 15.5 versus 57.9 % ± 17.1; *p* = 0.0016). Moreover, *TREM2* mRNA levels in the AD hippocampus correlated with enrichment in 5hmC at the *TREM2* gene body (*r* = 0.771; *p* = 0.005).

**Conclusions:**

*TREM2* mRNA levels are increased in the human hippocampus in AD cases compared to controls. DNA methylation, and particularly 5hmC, may be involved in regulating *TREM2* mRNA expression in the AD brain. Further studies are guaranteed to investigate in depth the role of 5hmC in AD and other neurodegenerative disorders.

**Electronic supplementary material:**

The online version of this article (doi:10.1186/s13148-016-0202-9) contains supplementary material, which is available to authorized users.

## Background

The triggering receptor expressed on myeloid cells 2 (*TREM2*) gene [EMBL: AB601768] encodes a transmembrane glycoprotein receptor that activates the innate immune response in macrophages and dendritic cells. In the brain, *TREM2* is expressed in microglia and it seems to promote phagocytosis of apoptotic neurons, cellular debris, and misfolded proteins by recognizing specific endogenous ligands on the surface of apoptotic cells [1–3]. At the same time, *TREM2* retards the inflammatory response by repressing microglial cytokine production [3]. Thus, *TREM2* seems critical to maintain brain homeostasis in response to tissue damage.

Lately, genome-wide association studies (GWAS) revealed *TREM2* gene variant rs75932628-T to be associated with Alzheimer’s disease (AD) and other neurodegenerative diseases, such as Parkinson’s disease, frontotemporal dementia and amyotrophic lateral sclerosis [4–7]. However, the mechanisms by which *TREM2* mutations might increase the risk of AD remain elusive. A recent study showed that loss of a single copy of *TREM2* significantly altered the morphological phenotype of β-amyloid plaque-associated microglia in the APPPS1-21 AD mouse model [8]. In the case of rs75932628-T variant, arginine to histidine substitution (R47H) may have a significant effect on the ligand binding affinity and reduce the phagocytic activity [9–12]. Specifically, *TREM2* is supposed to promote phagocytosis of Aβ42 peptides, preventing β-amyloid accumulation and downstream neurotoxic effects [13, 14]. Most recently, it was shown that R47H impairs detection of lipid ligands known to associate with fibrillar β-amyloid [15]. Therefore, impairment in clearance of Aβ42 and cellular debris may in part explain the increased risk of AD in carriers of *TREM2* gene variants [16].

On the other hand, the role of non-mutated *TREM2* in sporadic AD also needs further investigations. Notably, *TREM2* messenger RNA (mRNA) was upregulated in amyloid plaque-associated versus plaque-free brain tissue of aged APP23 mice, a transgenic AD mouse model [17]. Using another transgenic mouse model, *TREM2* was found to be overexpressed in microglia during disease progression [14]. *TREM2* expression has also been assessed in humans. According to a microarray-based expression study on *postmortem* brain samples from normal individuals, highest levels of *TREM2* mRNA were identified in the lobar white matter, substantia nigra, and medulla [18]. However, studies on *TREM2* expression in the AD human brain are scarce and controversial with some authors showing increased levels of *TREM2* in AD [19–21], while others reported downregulation of *TREM2* in the AD context [22].

Here, we investigated mRNA levels of *TREM2* in the human hippocampus in a cohort of neuropathologically defined “pure” AD cases and controls. Moreover, to assess epigenetic mechanisms potentially involved in regulating *TREM2* in AD, we profiled DNA methylation at different regulatory regions of the *TREM2* gene in the AD hippocampus.

## Results

### *TREM2* mRNA levels are upregulated in Alzheimer’s disease hippocampus

**Fig. 1:**
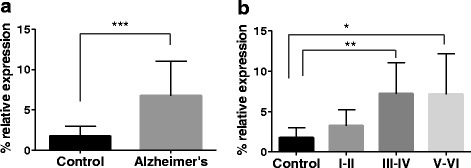
*TREM2* mRNA expression is increased in human hippocampus in Alzheimer’s disease (AD). **a** The graph shows a significant 3.4-fold increase in *TREM2* mRNA levels in AD hippocampal samples compared to control hippocampal samples. **b** *TREM2* mRNA expression increases across AD stages, as shown when *TREM2* mRNA expression levels are sorted by Braak & Braak stages. *Bars* represent percentage of *TREM2* mRNA expression relative to the geometric mean of *HPRT* and *ACTB* housekeeping genes expression. *Vertical lines* represent the standard error of the mean.*p value <0.05; **p value <0.005; ***p value <0.0005

We first measured *TREM2* mRNA levels in hippocampal samples from Alzheimer’s disease (AD) cases and controls by RT-qPCR. Four samples did not pass the RNA quality threshold so were not included in the experiments (see *TREM2 mRNA expression analysis* in the “Methods” section). Eventually, 26 AD cases were compared to 12 controls. None of the subjects included in the study was carrying the *TREM2* rs75932628-T variant, in accordance with the low frequency of the variant allele in the European ancestry population [7]. A 3.4-fold increase in *TREM2* mRNA levels was observed in the hippocampus of AD cases compared to controls (mean ± SD mRNA levels in AD versus controls: 6.65 ± 4.30 % versus 1.73 ± 1.24 %; *p* = 1.1E-05) (Fig. 1a). Next, a disease-staging analysis was conducted to investigate changes of *TREM2* mRNA levels considering AD severity. We found that *TREM2* mRNA levels significantly increased across AD stages (*p* = 0.003; Fig. 1b). Games-Howell post hoc analysis showed that *TREM2* mRNA expression was significantly different between control and Braak stages III–IV (*p* = 0.003) and between control and Braak stages V–VI (*p* = 0.035) (Fig. 1b).

Then, a logistic regression model was designed to identify adjusted estimates of the association of *TREM2* mRNA levels with AD status (control = 0; AD = 1). Age and gender were included into the model in order to adjust for potentially confounding variables, since there were significant age differences between the control and AD group and a statistical trend was found towards higher proportion of male in the control group (see *Human brain samples and neuropathological examination* in the “Methods” section). As shown in Table 1, *TREM2* mRNA expression remained as an independent predictor of AD status after adjusting for age and gender (*p* = 0.026) (Table 1).

**Table 1:** Adjusted logistic regression model to predict AD status

Variable	B	Wald	*p* value	OR	95 % CI inferior	95 % CI superior
*TREM2* mRNA levels	0.709	4.964	0.026*	2.031	1.089	3.789
Gender (female)	0.369	0.108	0.742	1.446	0.161	13.015
Age <65 yo	1.737	2.383	0.123	0.176	0.019	1.597
Constant	1.035	0.572	0.45	0.355	–	–

Alzheimer status (control = 0; AD = 1) was considered as the dependent variable, and *TREM2* mRNA expression levels, gender, and age were included as covariates. *B* regression coefficient, *OR* odds ratio, *yo* years old, *p value <0.05

We further tested if differences in *TREM2* mRNA levels resulted from an increase in a particular type of *TREM2* splice variant. Two different types of *TREM2* transcripts were identified in the Reference Sequence (RefSeq) database, i.e., *TREM2* transcript variant 1 (NM_018965) and *TREM2* transcript variant 2 (NM_001271821) (Additional file 1: Figure S1.A). Next, two sets of primers that separately amplify each *TREM2* splice variant were designed (Additional file 1: Table S1 and Figure S1.A). Interestingly, we found that mRNA levels of each of both transcripts were significantly higher in the hippocampus from AD patients compared to controls (Additional file 1: Figure S1.B). Nonetheless, the most prominent splice variant in the hippocampal tissue was *TREM2* transcript variant 1 (NM_018965), suggesting that the majority of mRNA changes may be accounted for by an increase in that particular splice variant.

### *TREM2* mRNA levels positively correlate with β-amyloid and p-tau burden

Next, *TREM2* mRNA expression was examined in more detail according to the neuropathological AD-related changes measured and recorded from the hippocampus sections. The amyloid plaque score (APS) and average area of both β-amyloid deposition and hyperphosphorylated tau (p-tau) were quantitatively measured as described in the “Methods” section by using ImageJ software [23] (Additional file 1: Figure S2) in the whole set of 30 AD cases and 12 controls included in the study. Interestingly, a significant association between APS and *TREM2* mRNA levels was found in the human hippocampus (*r* = 0.593, *p* = 1.4E-04). In addition, the average area of β-amyloid burden in the hippocampus was also correlated with *TREM2* mRNA levels (*r* = 0.602, *p* = 1.0E-04), suggesting that *TREM2* mRNA expression parallels the molecular changes induced by β-amyloid deposition. Since we had found an increased *TREM2* mRNA expression across Braak stages, a positive correlation with p-tau deposits was expected. Indeed, the average area of p-tau deposition, which included neurofibrillary tangles, neuropil threads, and neuritic plaques, was positively correlated to *TREM2* mRNA levels (*r* = 0.509, *p* = 0.002).

### DNA methylation in the *TREM2* transciption start site (TSS)-associated region is increased in AD cases compared to controls

To further explore if differences in *TREM2* mRNA levels between AD cases and controls were related to DNA methylation changes, bisulfite cloning sequencing was conducted in a subset of six controls and 10 Alzheimer’s hippocampal samples. Bisulfite cloning sequencing assesses changes in DNA methylation which includes modifications in 5-methylcytosine (5meC) and 5-hydroxymethylcytosine (5hmC). There were no significant differences between control and AD groups regarding age (52 ± 27.9 years versus 74.8 ± 13.7; *p* value = 0.107) or gender (male percentage) (62.5 versus 62.5 %; *p* value = 1.0). Moreover, PMI was similar in both groups (7.1 ± 2.9 h versus 7.6 ± 5.6 h; *p* value = 0.821).

**Fig. 2:**
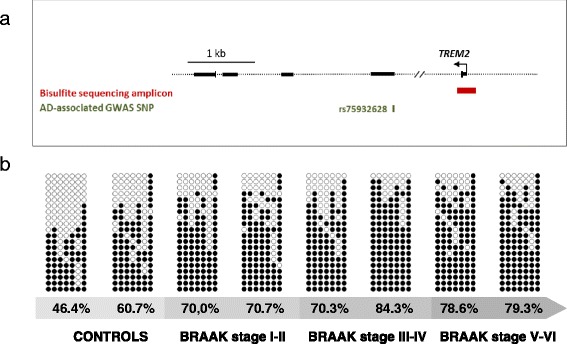
DNA methylation levels in the *TREM2* TSS-associated region in Alzheimer’s disease (AD) and control hippocampus. **a** The figure shows the genomic position of the amplicon surveyed by bisulfite cloning sequencing at the TSS-associated region of the *TREM2* gene. At the bottom of the figure, the GWAS-associated *TREM2* variant (rs75932628) position is also shown. **b** The picture shows the methylation pattern at CpG site resolution of *TREM2* TSS-associated region for different samples across AD progression. It is only an example of the bisulfite results for each group. Therefore, two individual subjects (samples) have been included per group. *Black and white circles* represent methylated and unmethylated cytosines, respectively. Each *column* symbolizes a unique CpG site in the examined amplicon, and each *line* represents an individual DNA clone. Global percentage of methylation for each analyzed sample (control or patient) at this particular amplicon is indicated at the bottom of each sample. *Meth Amp* methylation amplicon, *GWAS* genome-wide association studies

Percentage of DNA methylation at seven individual cytosine guanine dinucleotides (CpGs) in the TSS-associated region of *TREM2* gene (Fig. 2a) was measured by sequencing a minimum of 20 clones per individual. Then, average DNA methylation for the entire amplicon was calculated in each individual. Following that approach, a significant increase in DNA methylation at the TSS-associated region of *TREM2* gene was observed in the AD cases group compared to the control group (76.2 ± 15.5 % versus 57.9± 17.1 %; *p* = 0.0016) (Fig. 2b). All seven interrogated CpGs at the TSS-associated region showed a significant (*p* < 0.05) gain in DNA methylation in AD cases compared to controls (Table 2). After Bonferroni correction, differences between AD and controls in the average DNA methylation for the whole amplicon and DNA methylation in the first CpG of the amplicon (CpG at position 24) remained significant (adjusted *p* value = 0.00625; Table 2). Interestingly, this result did not follow the classical model of epigenetic regulation where gain in methylation in the promoter region correlates with a decrease in gene expression. To improve the specificity of our assay, we used a negative control by bisulfite sequencing an amplicon located at −874 bp relative to *TREM2* transcription start site (Additional file 1: Figure S3). There were no statistically significant differences between AD and control group (90.7 ± 3.9 versus 92.5 ± 6.45; *p* value = 0.664) for this particular region. We also wanted to test the relationship between average DNA methylation and mRNA expression for the *TREM2* gene. No correlation was found between DNA methylation percentage measured by bisulfite sequencing and *TREM2* mRNA levels in our set of samples (*r* = 0.391, *p* value = 0.134).

**Table 2:** Averaged methylation of each CpG site in control and AD groups

CpG position	24	65	129	175	236	269	471	Total	SD
MR control group. %	60.4	68.10	47.9	50.00	62.5	31.2	85.4	57.91	17.1
MR AD group. %	83.7	85.4	66.7	70.7	80.5	49.2	96.7	76.16	15.5
Gain in methylation. %	23.3	17.3	18.8	20.7	18.00	18.00	11.3	18.25	-
*p* value	0.0015*	0.0163	0.0353	0.0130	0.0180	0.0400	0.0121	0.0016*	-

*CpG position* denotes the position of the dinucleotide CpG within the amplicon. *Total* denotes the averaged methylation percentage for the whole amplicon. *MR* methylation ratio, *AD* Alzheimer’s disease, *SD* standard deviation. *significant after Bonferroni correction (adjusted *p* value = 0.00625)

### 5hmC enrichment in the *TREM2* gene body correlates with *TREM2* mRNA levels in the AD hippocampus

**Fig. 3:**
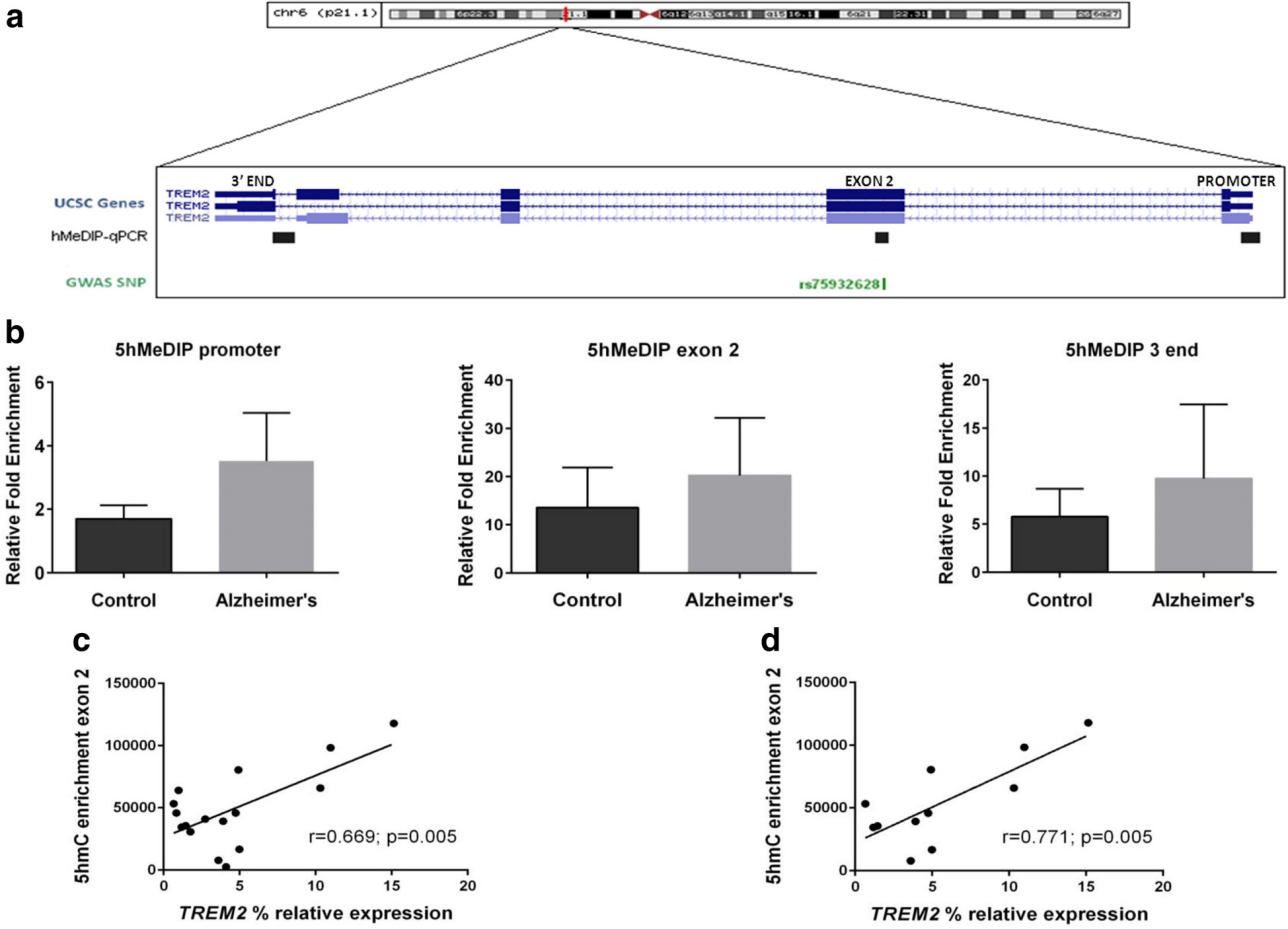
5-Hydroxymethylated DNA profiling in Alzheimer’s versus control hippocampus. **a** The figure shows the genomic locations of the amplicons (*black bars*) designed to assess the 5hmC enrichment at different regions of *TREM2* by 5-hydroxymethylated DNA immunoprecipitation combined with RT-qPCR. UCSC Genes line denotes *TREM2* gene predictions from the USCS track at the UCSC Genome Browser. hMeDIP-qPCR line shows the examined amplicons at the 3′ end of the gene (*left*), the gene body at exon 2 (*middle*), and the TSS-associated region (*right*). The GWAS SNP line shows the genomic position of the *TREM2* variant (rs75932628) which is included in the amplicon at exon 2. **b** 5hMeDIP experiments, using an antibody against 5-hydroxymethylcytosine (5-hmC), revealed no differences in levels of 5-hydroymethylated DNA on the TSS-associated region (2.6-fold enrichment, *p* = 0.130) (*left*), and the gene body in exon 2 (1.5-fold enrichment, *p* = 0.383) (*middle*) and the 3′ end (1.7-fold enrichment, *p* = 0.633) (*right*) of *TREM2* in the hippocampus samples of AD patients compared to controls. The graphs **c** and **d** show statistically significant positive correlation between 5hmC enrichment in the *TREM2* exon2 region for the whole set of samples (**c**) and only for the AD-affected hippocampus (**d**)

Next, we asked whether DNA methylation differences observed in AD cases were indeed the result of 5hmC changes, as higher 5hmC levels at specific sites have been related to increased gene expression [24, 25]. Since bisulfite cloning sequencing is unable to distinguish between 5mC and 5hmC, we performed 5-hydroymethylated DNA immunoprecipitation (5hMeDIP) to selectively enrich DNA fragments containing 5hmC, followed by quantification by RT-qPCR, in a group of 12 AD cases and 5 controls. Following that procedure, we were able to estimate 5hmC levels not only at the TSS-associated region but also at exon 2 and at the 3′ end of the gene. Noticeably, amplicon covering exon 2 included the rs75932628-T variant associated with AD in GWAS (Fig. 3a). No statistically significant differences in 5hmC enrichment were found between groups at *TREM2* TSS-associated region (IP/INPUT fold change enrichment ± SD in AD versus controls: 3.52 ± 5.48 versus 1.69 ± 0.95; *p* value = 0.130), exon 2 (AD versus controls: 18.70 ± 12.73 versus 16.73 ± 5.14; *p* value = 0.383) and 3′end (AD versus controls: 9.76 ± 7.72 versus 5.77 ± 2.91; *p* value = 0.633) (Fig. 3b).

Interestingly, a significant positive correlation was found between 5hmC enrichment in exon 2 and *TREM2* mRNA levels (*r* = 0.669; *p* = 0.005) (Fig. 3c) for the whole set of samples. When considering only the AD hippocampal samples, correlation between 5hmC enrichment in exon 2 and *TREM2* mRNA expression was found to be even stronger (*r* = 0.771; *p* = 0.005) (Fig. 3d).

## Discussion

An important finding of this study is that *TREM2* mRNA is significantly upregulated in the human hippocampus in Alzheimer’s disease (AD) and correlates with AD-related neuropathological changes. Furthermore, we show that *TREM2* mRNA levels in the human hippocampus positively correlates with 5-hydroxymethylcytosine (5hmC) enrichment in the *TREM2* gene body, suggesting that 5hmC may be involved in regulating *TREM2* in the AD context.

Our results on *TREM2* mRNA expression are in high concordance with previous reports conducted in *postmortem* human brain samples that had showed upregulation of *TREM2* in the inferior temporal cortex in sporadic AD cases [19] and a trend for *TREM2* to be increased in the hippocampus of AD cases [20]. Accordingly, we found an increase in *TREM2* mRNA levels in the AD hippocampus that indeed did reach consistent statistical significance in our set of samples. Nevertheless, replication of our result in a wider and independent cohort would be of most interest to support this finding.

Higher levels of *TREM2* mRNA might reflect the activation of microglial cells in the AD-affected brain, a phenomenon well known to occur in neurodegenerative diseases [26]. In fact, one explanation for high *TREM2* mRNA levels in AD cases would be either that microglia increases *TREM2* mRNA expression as an attempt to maintain the homeostasis after brain damage or a distinct increase in the proportion of reactive microglia in AD hippocampus, or even a combination of both. Further assays to measure proportion of cell population in AD and control cases, as with fluorescence-activated cell sorting (FACS), would be most helpful to address this issue. Whatever the case, it is clear that neuroprotective mechanisms fail to provide enough protection, given the ominous AD clinical course. In the context of *TREM2* mutations, phagocytic function of *TREM2* would be impaired, compromising even more the repair potential of neuroprotective mechanisms. In agreement with the latter idea, rs75932628-T variant in *TREM2* accelerates the AD clinical course reducing the age at onset [27] and significantly shortening the disease duration [26].

Interestingly, *TREM2* mRNA levels were positively correlated with both β-amyloid and p-tau burden in our study, as it would be expected of a protective mechanism trying, but not achieving, to repair brain damage. This positive correlation is in line with previous research conducted in AD mouse models that revealed upregulation of *TREM2* mRNA in the amyloid plaque boundaries [17], and those performed on human brain samples that showed positive correlation between *TREM2* mRNA levels and p-tau burden in mid-temporal gyrus from AD patients [21]. Taken together, these data suggest that *TREM2* overexpression could be a crucial but insufficient attempt to repair the brain tissue, as others have also proposed [9, 26].

The putative relationship between *TREM2* mRNA expression and microglia activation in AD does not preclude epigenetic involvement in regulating *TREM2*. Indeed, the methylation pattern observed for *TREM2* in our set of samples could be part of the epigenetic signature of activated microglia in the AD brain. Actually, epigenetic modulation of microglia phenotypes has been described in other neurodegenerative diseases, such as Parkinson disease [28]. However, epigenetic mechanisms involved in microglia activation still need further research. A limitation of our study is that our bisulfite sequencing analysis was performed in whole brain tissue, which indeed constitutes a mixed cell population. Hence, our bisulfite analysis is not specific for microglial cells, and epigenetic results may be biased by this cell heterogeneity, as epigenetic regulation is usually cell-type specific.

An interesting aspect of our finding is that higher levels of methylation in the *TREM2* TSS-associated region parallel higher levels of mRNA expression. Classically, methylation of CpG islands located in promoter regions leads to silencing of gene expression. However, this rule does not fully apply to non-CpG island promoters, as it would be the case of the *TREM2* TSS-associated region, which lacks a CpG island (Fig. 2a). The role of DNA methylation in non-CpG island promoters is under investigation, and methylation of poor-CpG promoters does not preclude their activity [29]. Importantly, a positive correlation between promoter methylation and gene expression has also been described in other neurodegenerative conditions, such as Huntington disease [30]. Furthermore, cumulative evidence from recent studies suggests that regulation of gene expression by DNA methylation may be much more complex than the classical model description [31], and new epigenetic marks have been described, such as 5hmC, that seems to work differently from 5-methylcytosine (5mC) in controlling gene expression.

The 5hmC content varies drastically among different mammalian tissues, with the highest levels being found in the brain [32–34]. Moreover, the amount of 5hmC globally increases during normal brain aging, particularly in the hippocampus [35, 36]. Interestingly, global 5hmC levels are higher in human hippocampus and parahippocampal gyrus in AD subjects compared to controls [25]. Furthermore, in *postmortem* human samples, global 5hmC levels positively correlate with β-amyloid and tau loads, in the frontal and temporal gyrus [37]. Regarding gene expression, it has been described that 5hmC may play a dual role in regulating both transcriptional activation and repression [38]. In the brain tissue, 5hmC is enriched in the gene bodies of actively transcribed genes, where it is directly correlated with expression levels of the corresponding genes [25, 39]. Consistent with the latter statement, we found in the AD hippocampus a significant and positive correlation between 5hmC enrichment in the *TREM2* gene body at exon 2 and *TREM2* mRNA levels. Noticeably, the amplified region within exon 2 harbors the rs75932628-T variant previously associated to AD by GWAS. So, a cis-regulatory effect on 5hmC levels would be worthy to be examined in future studies.

## Conclusions

In summary, this study provides evidence that *TREM2* mRNA is upregulated in the human hippocampus affected by AD. Our findings also suggest that 5hmC may play a role in regulating *TREM2* mRNA expression, a gene related to the AD pathogenesis. Further studies are guaranteed to investigate in depth the regulatory role of 5hmC in AD and other neurodegenerative disorders.

## Methods

### Human brain samples and neuropathological examination

We conducted an observational, case-control study comparing *postmortem* hippocampal samples from AD patients and control hippocampus. Frozen *postmortem* hippocampal samples from 30 Alzheimer’s disease (AD) cases and 12 controls were provided by the Navarrabiomed Brain Bank. After death, half brain specimens from donors were cryopreserved at −80 °C.

Neuropathological examination was completed following the usual recommendations [40]. Assessment of β-amyloid deposit was carried out by immunohistochemical staining of paraffin-embedded sections (3- to 5-μm thick) with a mouse monoclonal (S6F/3D) anti β-amyloid antibody (Leica Biosystems Newcastle Ltd, Newcastle upon Tyne, UK). Evaluation of neurofibrillary pathology was performed with a mouse monoclonal antibody anti-human PHF-TAU, clone AT-8, (Tau AT8) (Innogenetics, Gent, Belgium), which identifies hyperphosphorylated tau (p-tau) [41]. The reaction product was visualized using an automated slide immunostainer (Leica Bond Max) with Bond Polymer Refine Detection (Leica Biosystems Newcastle Ltd).

Subjects received diagnosis of pathophysiologically proved AD dementia according to the updated National Institute on Aging-Alzheimer’s Association guidelines [42]. Subjects were further classified, based on neurofibrillary pathology, as follows: control (*n* = 12); Braak stages I–II (*n* = 3); Braak stages III–IV (*n* = 17); and Braak stages V–VI (*n* = 10) [41, 43]. Importantly, to avoid spurious associations, those individuals showing coincident protein deposits different from p-tau or β-amyloid were not eligible for the study. This approach maximizes chances of finding true associations with AD, even though reducing the final sample size. Neuropathological and demographic features of subjects, including age, gender, and *postmortem* interval are listed in Additional file 1: Table S2. There were significant age differences between the control and AD group (50.7 ± 21.5 years versus 82.3 ± 11.3 years, *p* < 0.01), and a statistical trend was found towards higher proportion of male in the control group (66.7versus 34.5 %, *p* = 0.87). The postmortem interval (PMI) was not significantly different between groups (8.2 ± 4.2 h in the control group versus 7.9 ± 7.1 h in the AD group; *p* = 0.91).

### Ethics, consent, and permissions

The Ethics Committee of the “Complejo Hospitalario de Navarra” approved the use of human subjects for this study (Pyto 90/2014). Written informed consent was obtained from all subjects or next of kin.

### Genotyping of *TREM2* variant rs75932628-T

We wanted to assess whether any subject included in our study was harboring the *TREM2* rare variant rs75932628-T. To that end, we genotyped the *TREM2* variant rs75932628-T as it has been previously described [44]. In brief, a PCR reaction was carried out to amplify a 172 base pair (bp) amplicon spanning the rs75932628 variant. Next, a restriction fragment length polymorphism analysis was performed by using the restriction enzyme *HhaI* (Thermo Fisher Scientific Inc., Waltham, MA, USA). The change from C to T in rs75932628 results in loss of the *HhaI* restriction enzyme site. When separated by electrophoresis, digested PCR fragments from homozygous wild-type subjects show two bands of 89- and 84-bp length, whereas heterozygous subjects harboring *TREM2* variant rs75932628-T display an additional 172 bp band, corresponding to the allele that has lost the restriction enzyme site.

### *TREM2* mRNA expression analysis

Total RNA was isolated from hippocampus homogenates using RNeasy Lipid Tissue Mini kit (QIAGEN, Redwood City, CA, USA), following manufacturer’s instructions. Genomic DNA was removed with a recombinant DNase (TURBO DNA-free™ Kit, Ambion, Inc., Austin, TX, USA). RNA integrity was checked by 1.25 % agarose gel electrophoresis under denaturing conditions. Concentration and purity of RNA were both evaluated with NanoDrop spectrophotometer. Only those RNA samples showing 260 nm/280 nm absorbance ratios between 1.8 and 2.2 and 260 nm/230 nm absorbance ratios higher than 1.8 were considered high enough quality to be included in the study. Complementary DNA (cDNA) was reverse transcribed from 1500 ng total RNA per sample with SuperScript® III First-Strand Synthesis Reverse Transcriptase (Invitrogen, Carlsbad, CA, USA) and primed with a mixture of oligo-d (T) and random primers.

Real-time qPCR (RT-qPCR) reactions were performed in triplicate for each sample using Power SYBR® Green PCR Master Mix (Invitrogen, Carlsbad, CA, USA) in a 7300 real-time PCR System (Applied Biosystems, Foster City, CA, USA). Experiments were repeated twice within independent cDNA sets. Sequences of primer pair were designed by using real-time PCR tool (IDT, Coralville, IA, USA) and are listed in Additional file 1: Table S1. The PCR amplicon sizes range from 116 to 184 bp. All the primers used in RT-qPCR assays were tested for specificity. During designing the primer sets, a Basic Local Alignment Search Tool (BLAST) versus entire mRNA RefSeq database was performed to verify the correct and unique alignment of the fragment. Moreover, dissociation curves were obtained for each assay to check that curves show a single peak with no shoulder. After amplification, we also checked that RT-qPCR reaction had generated a single amplicon of the correct size by performing agarose gel electrophoresis.

The thermal cycling conditions consisted of an initial denaturation step at 95 °C for 10 min followed by 40 cycles of 15 s at 95 °C and 1 min at 60 °C. The geometric mean of *ACTB* and *HPRT* housekeeping genes was used to normalize *TREM2* mRNA levels [45], and non-template reactions were included as negative controls in each run. Relative expression level of *TREM2* mRNA in a particular sample was calculated by the delta delta-CT method, as previously described [46].

### Quantitative assessment of β-amyloid and p-tau deposits in brain tissues

In order to quantitatively assess the β-amyloid and p-tau burden for further statistical analysis, we applied a method to quantify protein deposits. This method generates a numeric measurement that reflects the extent of β-amyloid and p-tau deposition. In the case of p-tau, not only neurofibrillary tangles but also neuritic plaques and neuropil threads are taken into account (Additional file 1: Figure S2). Sections of the hippocampus were examined after performing immunostaining with anti β-amyloid and anti-p-tau antibodies as described above in *Human brain samples and neuropathological examination*. Three pictures were obtained for each immunostained section by using an Olympus BX51 microscope at ×10 magnification power. Focal deposit of β-amyloid, as described by Braak & Braak (neuritic, immature, and compact plaque) [43], was manually determined and was further edited and analyzed with the ImageJ software (figure e-1). Then, β-amyloid plaque count, referred to as amyloid plaque score (APS) (Additional file 1: Figure S2) and total area of β-amyloid deposition were automatically measured by ImageJ and averaged for each section. Regarding p-tau deposit, representative pictures were analyzed with ImageJ software in order to obtain an average quantitative measure of the global p-tau deposit for each section (Additional file 1: Figure S2).

### Methylation in the TSS-associated region of *TREM2* by bisulfite cloning sequencing

Genomic DNA was isolated from hippocampal tissue by standard methods [47]. Next, 500 ng of genomic DNA was bisulfite converted using the EpiTect Bisulfite Kit (QIAGEN, Redwood City, CA, USA) according to the manufacturer’s instructions. An amplicon spanning the TSS-associated region of *TREM2* was amplified by PCR (Fig. 2a). Primer pair sequences were designed by MethPrimer [48] and are listed in Additional file 1: Table S1. Genomic map of the amplicon was drawn using the UCSC Genome Browser [49]. Next, PCR products were cloned using the TopoTA Cloning System (Invitrogen, Carlsbad, CA, USA), and a minimum of 20 independent clones were sequenced for each examined subject by Sanger sequencing [50].

### Determination of 5hmC in hippocampus samples

We evaluate 5-hydroxymethycytosine (5hmC) by performing 5-hydroxymethylated DNA immunoprecipitation combined with RT-qPCR (5hMeDIP-RT-qPCR). Genomic DNA (1500 ng) was sonicated to obtain fragments of 150–500 base pair length that were assessed by 1 % agarose gel electrophoretic analysis. Next, DNA containing 5hmC was enriched using the EpiQuik Hydroxymethylated DNA Immunoprecipitation (hMeDIP) Kit (Epigentek, Farmingdale, NY, USA), according to manufacturer’s recommendations. Primer pair sequences are listed in Additional file 1: Table S1. Serial dilutions of samples were performed to determine amplification efficiency for each primer pair.

Real-time qPCR (RT-qPCR) reactions were performed in triplicate for each sample using Power SYBR® Green PCR Master Mix (Invitrogen, Carlsbad, CA, USA) in a 7300 real-time PCR System (Applied Biosystems, Foster City, CA, USA). The PCR amplicon sizes range from 97 to 108 bp. All the primers used in RT-qPCR assays were tested for specificity as indicated above. The amplification reaction program included an initial step of 95 °C for 10 min, followed by 40 cycles of 95 °C for 15 s, 60 °C for 1 min, and a final melt curve analysis step. DNA control from the kit was included in the experiments. Fold enrichment was calculated as the ratio of amplification efficiency of each immunoprecipitated sample over the efficiency of non-immune IgG as of the following the formula: 2^dCt^, where dCt = Ct^INPUT^ – Ct^hMeDIP^, and further normalized to the lowest detectable sample.

### Statistical data analysis

Statistical analysis was performed with SPSS software version 21.0 (IBM, Inc., USA). Before performing differential analysis, we checked that all continuous variables showed a normal distribution, as per one-sample Kolgomorov-Smirnov test and the normal quantil-quantil (QQ) plots. Summarized data from continuous variables were expressed as mean ± standard deviation. Significance level for all comparisons was set at *p* value <0.05. Statistical significance for intergroup differences was assessed by the Pearson’s chi-square test for categorical variables and the independent sample *t* test for continuous variables. After finding that variances were unequal by Levene’s test, one-way analysis of variance (ANOVA) followed by Games-Howell post hoc analysis was used to analyze differences in the expression levels of *TREM2* mRNA between stage groups. A logistic regression model (ENTER method) was fit to assess the independent association of *TREM2* mRNA levels with AD status, using gender and age as covariates. The Pearson product-moment correlation coefficient analysis was used to correlate *TREM2* mRNA expression levels with neuropathological changes. Methylation ratio was calculated as the ratio of methylated CpGs over the total number of CpGs assessed for each CpG site. Gain in methylation was calculated as the subtraction of percentage of methylation between the AD group and control group. Although methylation is not completely independent for each CpG site within the same amplicon, a conservative approach to avoid false positive results were used by applying Bonferroni correction. Difference between two bisulfite sequence groups was evaluated with Mann-Whitney *U* test. GraphPad Prism version 6.00 for Windows (GraphPad Software, La Jolla, CA, USA) was used to draw the graphs except for methylation figures that were drawn by QUMA software [51].

## Additional file

Additional file 1: Contains supplemental **Figure S1** (Both TREM2 splice variants showed increased mRNA expression in human hippocampus in Alzheimer’s disease), **Figure S2** (Beta-amyloid and tau protein measurement in hippocampal sections) and **Figure S3** (A negative control upstream the TREM2 promoter was surveyed by bisulfite cloning sequencing) along with supplemental **Table S1** (primers) and **Table S2** (subjects characteristics). (PDF 656 kb)

## Abbreviations

5hmC: 5-hydroxymethycytosine
5hMeDIP: 5-hydroymethylated DNA immunoprecipitation
5mC: 5-methylcytosine
AD: Alzheimer’s disease
APS: amyloid plaque score
CpGs: cytosine guanine dinucleotides
GWAS: genome-wide association studies
p-tau: hyperphosphorylated tau
RT-qPCR: real-time qPCR
*TREM2*: triggering receptor expressed on myeloid cells 2
