# Supplementary material for: TREM2 upregulation correlates with 5-hydroxymethycytosine enrichment in Alzheimer’s disease hippocampus
Source: Clin Epigenetics. 2016 Apr 5;8:37. doi: 10.1186/s13148-016-0202-9 (PMC4820985; doi:10.1186/s13148-016-0202-9)
Supplement: Additional file 1: — Contains supplemental Figure S1 (Both TREM2 splice variants showed increased mRNA expression in human hippocampus in Alzheimer’s disease), Figure S2 (Beta-amyloid and tau protein measurement in hippocampal sections) and Figure S3 (A negative control upstream the TREM2 promoter was surveyed by bisulfite cloning sequencing) along with supplemental Table S1 (primers) and Table S2 (subjects characteristics). (PDF 656 kb) [file 13148_2016_202_MOESM1_ESM.pdf]

Additional File 1

Supplemental Figure S1

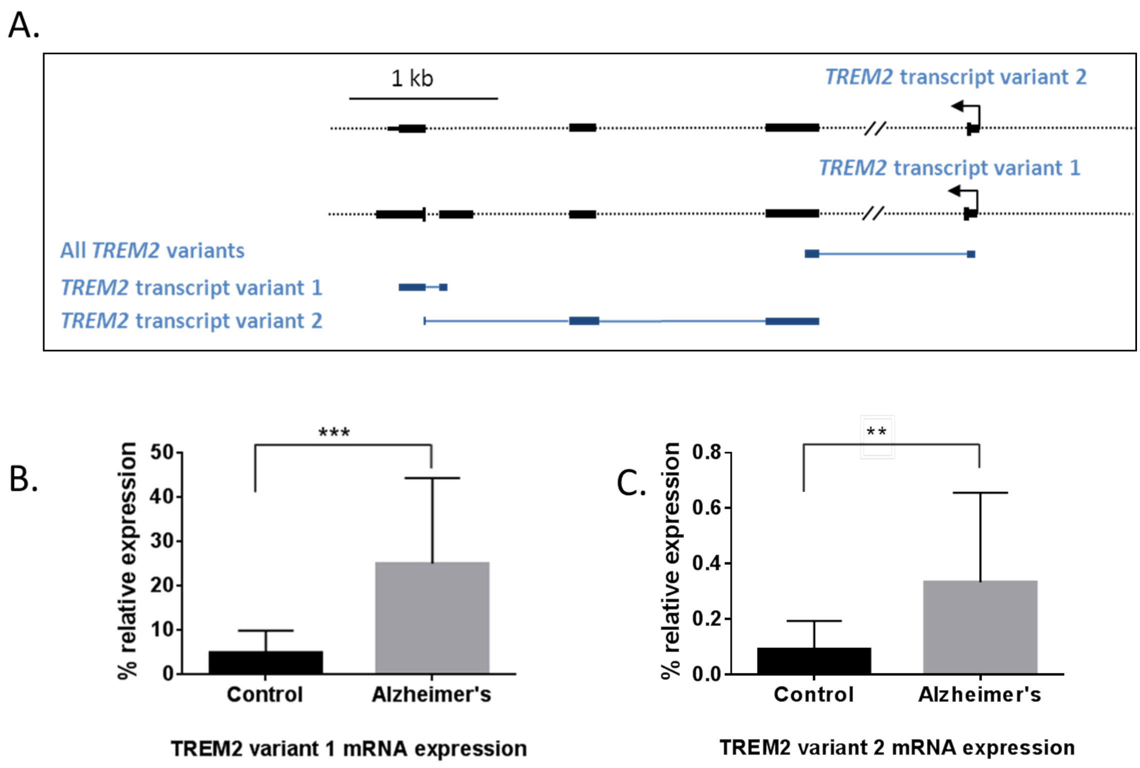

Both *TREM2* splice variants showed increased mRNA expression in human hippocampus in Alzheimer's disease (AD). A. The picture shows the map of the two different *TREM2* transcript variants*, TREM2* transcript variant 1 (NM_018965) and *TREM2* transcript variant 2 (NM_001271821). Black boxes represent exons and arrows represent transcription start sites. At the bottom lines, three different products of qPCR have been separately drawn, depending on the type of transcripts that are being amplified in the same reaction. B. The graph shows a significant increase in mRNA levels of *TREM2* transcript variant 1 in AD hippocampal samples compared to control hippocampal samples. C. mRNA levels of TREM2 transcript variant 2 are also significantly increased in AD hippocampal samples compared to control hippocampal samples. Boxes represent percentage of *TREM2* expression relative to the geometric mean of *HPRT* and *ACTB* housekeeping genes expression. Bars represent the standard error of the mean. ** p-value<0.005; ***p-value<0.0005

Supplemental Figure S2

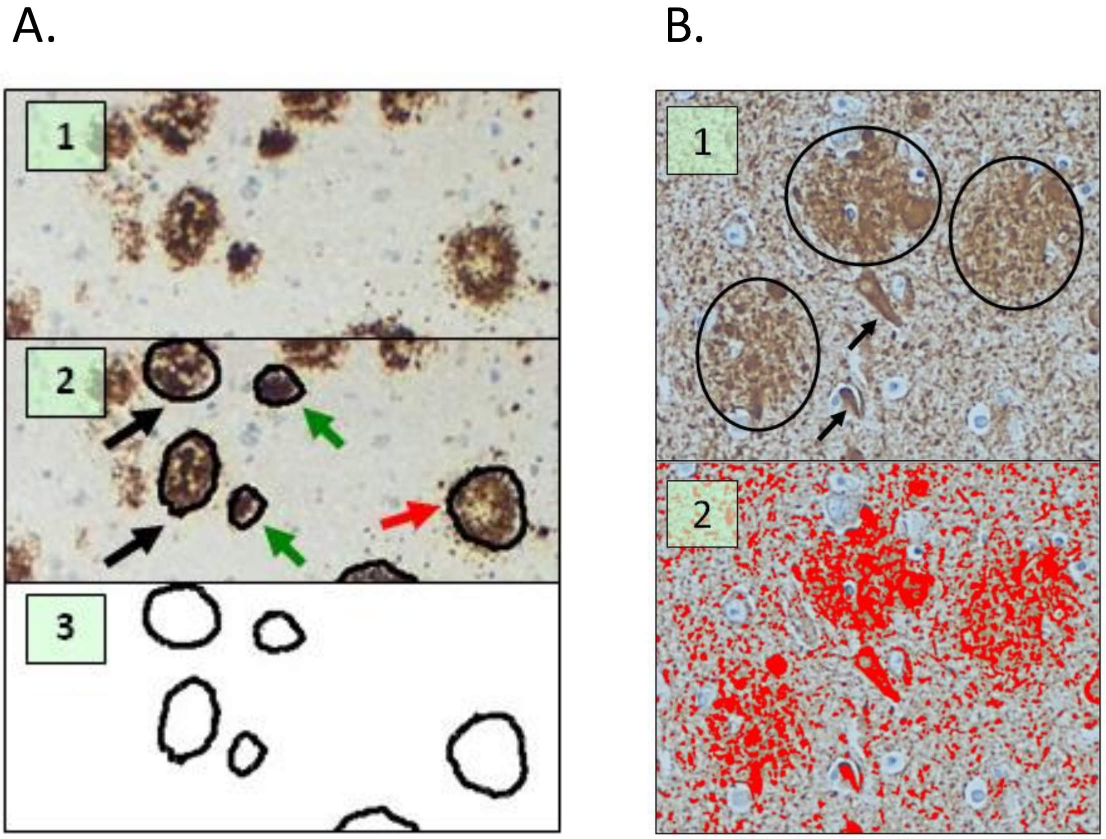

Beta-amyloid and tau protein measurement in hippocampal sections. A. The figure illustrates the method to measure amyloid deposits by using the ImageJ software. A1. Original figure of amyloid immunostained section obtained at 10x. A2. Selection of the different focal deposits patterns: neuritic plaques (black arrows), compact plaques (green arrows) and immature plaques (red arrow). A3. Analysis of the selected regions using the ImageJ software. B. Tau deposits measurement. B1. Tau immunostained section obtained at 10 x. The figure shows different deposits of tau protein: Neurofibrillary tangles (black arrows), neuritic plaques (black circles) and neuropil threads (background). B2: Selection of the whole deposit with the ImageJ software.

Supplemental Figure S3

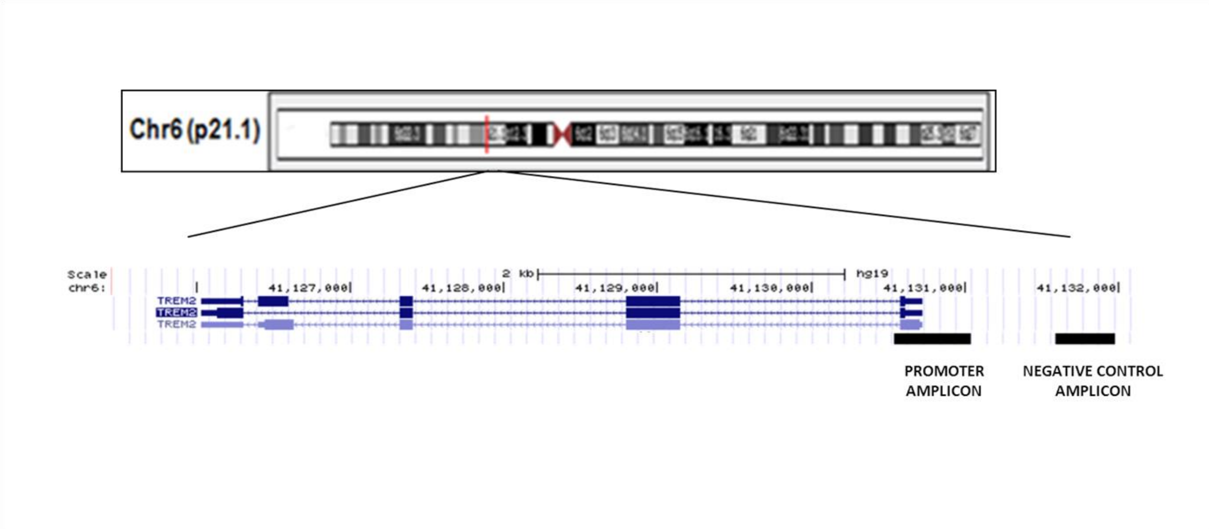

A negative control upstream the TREM2 promoter was surveyed by bisulfite cloning sequencing. The figure shows genomic position of the amplicons that were surveyed by bisulfite cloning sequencing: one amplicon spanning the promoter region of the *TREM2* gene (referred to as Promoter Amplicon) and a negative control amplicon located at -874 bp relative to *TREM2* transcription start site (referred to as Negative Control Amplicon).

Supplemental Table S1. Bisulfite and quantitative-PCR primers.

Identification	Amplified transcripts	PCR Purpose	Amplicon size	Tm	Forward Primer	Tm2	Reverse Primer	CpGs in amplicon
TREM2_qPCR	NM_018965, NM_001271821	qPCR	116 bp	61	CTGCTCATCTTACTCTTTGTCAC	62.3	CAGTGCTTCATGGAGTCATAGG	NA
TREM2_qPCR var 1	NM_018965	qPCR	145 bp	62.0	CAGGGTATCAGCTCCAAACTC	61.9	CCAGAGCAGAACAAGGAGTC	NA
TREM2_qPCR var 2	NM_001271821	qPCR	184 bp	55	ATGATGCGGGTCTCTACCAG	60	CTCTCAGCCCTGGAGATGCT	NA
TREM2_bis	NA	Bisulfite PCR	500 bp	54.89	GGGAGAGAAGGTATTATAGGGTA	52.79	CTCTTACAAAACTAAAACCAAAA	7
TREM2_OH_promoter	NA	5hMeDIP	97 bp	62.1	ATCTCGTCTTTCCCTTGAACTG	61.6	TCCTGACTATTGCTTAATCCCC	NA
TREM2_OH_exon 2	NA	5hMeDIP	108 bp	60.11	AAGGACAGCAGCCACAAGTT	59.67	TGACTCCATGAAGCACTGGG	NA
TREM2_OH_3' end	NA	5hMeDIP	98 bp	60.04	TGGAGAGCACAGCCATCAAG	60.04	GTGTCTCTCAGCCCTGCAAT	NA

The table shows the primer pairs used in the study.
bp: base pair; Tm: Melting Temperature.

Supplemental Table S2. Brain sample set analysed for *TREM2* expression.

No.	Diagnosis	Braak stage	APS	Age at death (years)	Gender	PMI (h)	Bisulfite cloning sequencing
1	Control	0	NA	61	male	8	no
2	Control	0	NA	81	male	10.5	yes
3	Control	0	NA	43	female	3	yes
4	Control	0	NA	88	male	9	yes
5	Control	0	NA	53	male	7	yes
6	Control	0	NA	41	male	3.5	no
7	Control	0	NA	28	male	6	yes
8	Control	0	NA	46	female	7	no
9	Control	0	NA	69	male	12	no
10	Control	0	NA	19	female	NA	yes
11	Control	0	NA	26	male	6.2	no
12	Control	0	NA	54	male	18	no
13	AD	I	0.00	60	male	15.3	yes
14	AD	I	2.00	85	male	3.2	no
15	AD	II	0.00	66	female	1.4	yes
16	AD	III	0	85	female	4.3	no
17	AD	III	0.00	88	female	33	no
18	AD	III	3.00	96	female	1.5	yes
19	AD	III	0.33	79	female	13	yes
20	AD	III	2.33	84	female	13	no
21	AD	III	2.00	98	female	23	no
22	AD	III	2.67	85	female	NA	no
23	AD	III	3.67	83	male	9	no
24	AD	III	6.67	69	female	4.3	no
25	AD	III-IV	1.00	81	female	9	no
26	AD	III-IV	6.67	98	female	3	no
27	AD	IV	5.00	88	male	3.5	yes
28	AD	IV	2.33	91	female	10	no
29	AD	IV	1.33	84	male	3.3	no
30	AD	IV	3.00	97	female	NA	no
31	AD	IV	1.33	78	male	5	no
32	AD	IV	1.33	90	female	3	no
33	AD	V	3.00	92	female	14	yes
34	AD	V	4.00	77	female	11	yes
35	AD	V	7.00	82	female	9	no
36	AD	V	8.00	91	male	5	no
37	AD	V	5.67	77	female	4	no
38	AD	VI	3.33	93	female	3	no
39	AD	VI	8.00	86	female	2.3	no
40	AD	VI	4.33	61	male	10	yes
41	AD	VI	9.67	70	male	2.35	yes
42	AD	VI	8.33	59	male	4	yes

The table shows characteristic of the samples included in the study (n=42).

No.: Number; APC: amyloid plaque score; h: hours; AD: Alzheimer's disease; PMI: *postmortem* interval.
